# Single-Center Experience with Swiss LithoClast^®^ Trilogy for Kidney Stones

**DOI:** 10.3390/diagnostics13081372

**Published:** 2023-04-08

**Authors:** Victor-Mihail Cauni, Florin Tanase, Bogdan Mihai, Gabriel-Petre Gorecki, Liana Ples, Romina-Marina Sima, Cristian Persu

**Affiliations:** 1 Department of Urology, Colentina Clinical Hospital, 020125 Bucharest, Romania; 2 Department of Anesthesia and Intensive Care, CF2 Clinical Hospital, 011464 Bucharest, Romania; 3 Faculty of Medicine, Titu Maiorescu University, 031593 Bucharest, Romania; 4 Department of Obstetrics and Gynecology, Carol Davila University of Medicine and Pharmacy, 050474 Bucharest, Romania; 5 “Bucur” Maternity, Saint John Hospital, 012361 Bucharest, Romania; 6 Department of Urology, Carol Davila University of Medicine and Pharmacy, 020021 Bucharest, Romania

**Keywords:** PCNL, mini perc, renal lithiasis, Lithoclast Trilogy, Lithoclast Master

## Abstract

Introduction: PCNL remains the gold standard for larger kidney stones. Reducing the operating time of PCNL and its complication rate seems to be the next logical step in optimizing this classical technique. To achieve these objectives, some new methods of lithotripsy emerge. We present the data of a single, high-volume, academic center with combined ultrasonic and ballistic lithotripsy in PCNL using the Swiss LithoClast^®^ Trilogy device. Materials and Methods: We designed a prospective, randomized study including patients who underwent PCNL or miniPerc with lithotripsy using the new EMS Lithoclast Trilogy or EMS Lithoclast Master. The procedure was carried out with all patients in prone position, by the same surgeon. The working channel size was 24 Fr–15.9 Fr. We evaluated the stones’ features, operative time, fragmentation time, complications, stone clearance rate and stone-free rate. Results: Our study included 59 patients, 38 females and 31 males, of an average age of 54.5 years old. The Trilogy group included 28 patients and the comparator included 31 patients. Urine culture was positive in seven cases which required seven days of antibiotics. The mean stone diameter was 35.6 mm with a mean Hounsfield unit (HU) of 710.1. The average number of stones was 2.08 (6 complete staghorn stones and 12 partial staghorn stones). A total of 13 patients presented a JJ stent (46.4%). We found a very significant difference in all the parameters favoring the Trilogy device. The most important result in our opinion is the probe active time, which was almost six times shorter in the Trilogy group. The stone clearance rate was about double in the Trilogy group, leading to shorter overall and intra-renal operating times. The overall complication rate was 17.9% in the Trilogy group and 23% in the Lithoclast Master group. The mean hemoglobin drop was 2.1 g/dL with a mean creatinine rise of 0.26 mg/dL. Conclusions: Swiss LithoClast^®^ Trilogy, a device combining ultrasonic and ballistic energy, is a safe and efficient method of lithotripsy for PCNL, proving statistically significant benefits over its predecesor. It can achieve the goal of reducing complication rates and operative times for PCNL.

## 1. Introduction

According to European and American guidelines for urolithiasis, percutaneous nephrolithotomy (PCNL) is the first-line treatment for kidney stones larger than 20 mm [1]. As a first-line therapy, the global experience with the technique is vast and the general interest in improving it is shown both by surgeons and industry. Any new advances have the objective of reducing the operative time and complication rate while maintaining or improving efficacy and safety [2]. Since the techniques of approaching the kidney and gaining access to the collecting system are both well-established, the focus now is on developing better and safer devices for stone breaking and improving vision inside the kidney. New intracorporeal lithotripsy devices are brought into the market with a promise to achieve these objectives. Traditionally, the most commonly used energy sources for lithotripsy were pneumatic (ballistic), ultrasonic and laser sources; each of these technologies has many advantages and disadvantages and not one of these has been distinguished as the best option for PCNL. Laser is less frequently used because of the higher cost incurred per procedure but also due to the lower efficacy for large stones compared to the other options. The use of laser became mainstream for flexible retrograde ureteroscopy, specifically RIRS (retrograde intra-renal surgery), in cases where the other options are not usable. Pneumatic lithotripters are very powerful, yet unexpensive when compared to other devices; nevertheless, their efficacy is limited precisely by their main strength, namely the serious amount of energy they deliver at every hit, which, in many cases, throws the stone or the fragments away from the line of fire, causing the surgeon to waste time moving in search of the fragments he is targeting. Ultrasonic devices rely on acoustic waves to generate high-frequency vibrations which destroy the stone, but this comes with a higher cost compared to pneumatic devises and has a slower fragmentation process.

In line with the modernization of lithotripsy tools, Swiss LithoClast^®^ Trilogy (EMS, Nyon, Switzerland) is one of the newest additions to the armamentarium of the modern urologist. This device uses the same probe as ultrasonic lithotripsy and ballistic lithotripsy use [3]. In the pneumatic mode, the Trilogy device uses a pneumatic probe to break up stones using mechanical energy. The ultrasonic mode uses acoustic waves to break up the stones. The Trilogy device also offers a combination mode in which both pneumatic and ultrasonic modes can be used together to increase the effectiveness of the lithotripsy procedure, a feature not previously available on the market. As an addition, the same probe can be used for suction also, and by using these three technologies in one probe, stone clearance is promised to be faster and more effective. Thus, the main advantage of Trilogy is not any new stone breaking technology, but the combination of the three most commonly used technologies into one single probe.

Considering the vast experience our center has with endourology in general and PCNL especially, many of the new technologies become available in our department shortly after they receive marketing clearance and we strive to evaluate every new device as thoroughly as possible and become early adopters of the devices that meet or exceed our expectations.

We hypothesized that the new device would at least significantly reduce the operative time and maybe improve the safety of PCNL consequently. The objective of our work was to analyze the results of combined lithotripsy using the Trilogy device and to evaluate its efficacy and safety by comparison to our previous lithotripsy workhorse, the Lithoclast Master, from the same manufacturer.

## 2. Materials and Methods

We designed a prospective, randomized study, aiming to analyze the results of the surgical treatment of kidney stones using EMS Swiss Lithoclast Trilogy by comparing the results of patients treated with this to those of patients treated with EMS Lithoclast Master, a device we have used to treat a very large number of cases in the past. Patients were randomized after the indication by PCNL/mini Perc was decided, based on our standard of care evaluation. Randomization was performed using a computerized random number generator (https://numbergenerator.org, accessed on 24 February 2023). Per our protocol, an odd number meant Lithoclast Master treatment while an even number meant Trilogy treatment. Ethical committee approval was obtained from the hospital’s Ethics Committee prior to the enrollment of any patient. Informed consent was obtained from all patients before undergoing surgery. Our standard informed consent form was used, since it does not mention the specific device used for stone fragmentation, but gives the patient information about the general and particular risks of percutaneous kidney surgery. 

The diagnostic protocol starts with a review of the patient’s medical history, with a focus on a history of lithiasis and comorbidities. Urinalysis and urine culture preparation is carried out in all cases per our protocol. A positive urine culture does not represent a contraindication to surgery, but antibiotics are started at once and, in some cases, the intervention is postponed for a few days. Imagistic evaluation starts with an ultrasonographic evaluation of the urinary tract and a KUB (kidney, ureter, and bladder radiography) procedure. A CT scan completes the functional evaluation of the urinary tract. Blood tests including coagulation evaluation are also standard for all our patients. The file is reviewed by the surgical staff and the indication for percutaneous surgery is decided and then discussed with the patient. The choice of the lithotripsy device remains the surgeon’s, except for this study in which randomization was used. We used Guy’s stone scores to predict the stone-free rate, as the only independent predictor after PCNL. Guy’s scores comprise four grades: grade I, where one stone is located in the middle or lower pole or renal pelvis if no anatomical abnormalities are present; grade II is defined as when there is a solitary stone in the upper pole of the kidney, several stones and a collecting system of a normal anatomy or a solitary stone and abnormal anatomy; grade III means there are multiple stones and the collecting system is of an abnormal anatomy or there is a partial staghorn calculus; grade IV is defined as the presence of a full staghorn stone or any kind of stone in patients with spinal cord injury or spina bifida.

Complications were evaluated using the Clavien Dindo classification, consisting of 5 main grades, as follows: grade I—any deviation from the normal postoperative evolution, with no need for any type of treatment; grade II—any complication which requires medical treatment or blood transfusion; grade III—complications which require surgical or radiological intervention; grade IV—a life-threatening complication requiring admission to intensive care; grade V—death of the patient.

For this study, we evaluated stone features, operative time, fragmentation time, stone clearance rate, stone-free rate and complications. Intraoperative complications, postoperative complications, hemoglobin drops, creatinine rises, and device malfunctions were recorded.

Stone size was measured by non-contrast CT in 2 diameters: a = longitudinal diameter and b = transverse diameter, in millimeters. Stone cross-sectional area was calculated using the ellipse area formula, π × a/2 × b/2. Stone density was documented in Hounsfield units (HU), and the number of stones and location were noted.

All PCNLs were performed in prone position, by the same surgeon, using fluoroscopy for access and monitoring. Although we also have significant experience with PCNL in the supine position, we decided to limit this study to the classic prone position in order to avoid introducing one more variable to the final analysis. The renal access channel size was 24 Fr for PCNL and 15.9 Fr for miniPCNL. Lithoclast Trilogy was used for lithotripsy using 3.4/3.9 mm probes for normal PCNL and 1.5 mm for miniPerc. In all cases, a nephrostomy tube was inserted and left in place at the end of the procedure. The total procedure time and nephroscopy time were registered using a stopwatch while the probe active time was registered by the medical devices. Both times were then noted in the patient’s file.

The stone-free rate was assessed at the end of the procedure by visual inspection and fluoroscopy, and during follow-up by ultrasound or CT. Stone clearance was calculated by dividing the stone area at the probe active time (mm^2^/min).

The Swiss Lithoclast Trilogy device is a new, powerful tool for intracorporeal lithotripsy, which has the main advantage of combining, within a single probe, ultrasonic and electromagnetic energy, thus creating a mix of ballistic and ultrasonic power, while also offering aspiration (suction) through the same probe. The ultrasonic mode uses high-frequency ultrasonic waves to break up kidney stones or other urinary tract stones. The ballistic mode uses a pneumatic probe to deliver mechanical energy to the stone, breaking it up into smaller pieces. The combination mode combines both ultrasonic and ballistic modes, offering a more effective way of breaking up larger or harder stones. The device comes with a range of probes of different sizes and shapes to allow the treatment of stones in different parts of the urinary tract and is designed with safety features to minimize the risk of injury or damage to surrounding tissues during the procedure [4]. Because the surgeon no longer needs to remove one probe and then insert the other and so on, the overall time of surgery decreases. On the other hand, no other device offers this mix of energy types, so the combined effect promises to be superior to anything else we have previously used. Still, the device allows the usage of one or another type of energy independently, while keeping the suction feature available all the time. The producer promises an overall 48% faster removal of stones compared with other devices with a similar reduction in the operative time [5]. 

Data analysis was performed with the IBM SPSS statistical software version 27, by a professional in our statistics department. Two sample *t*-test with pooled variance was used for comparison between the two groups.

## 3. Results

**Figure 1:**
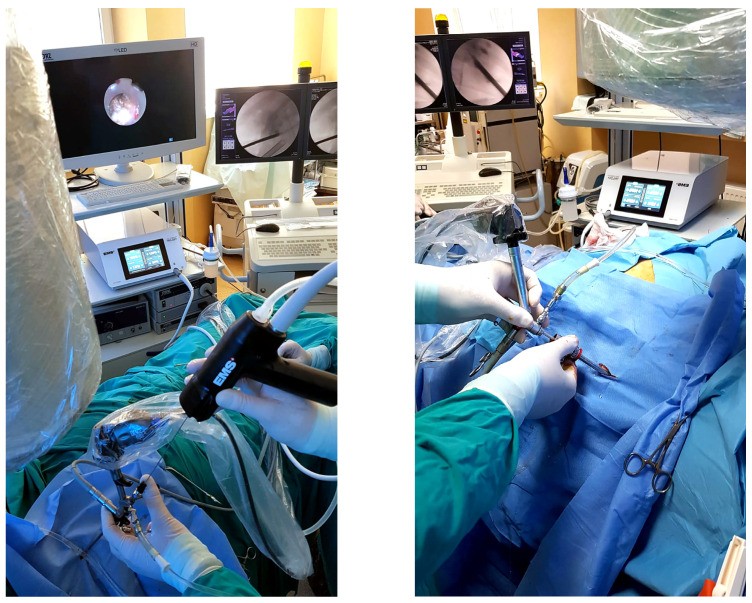
Intraoperative aspects of the Trilogy device.

**Figure 2:**
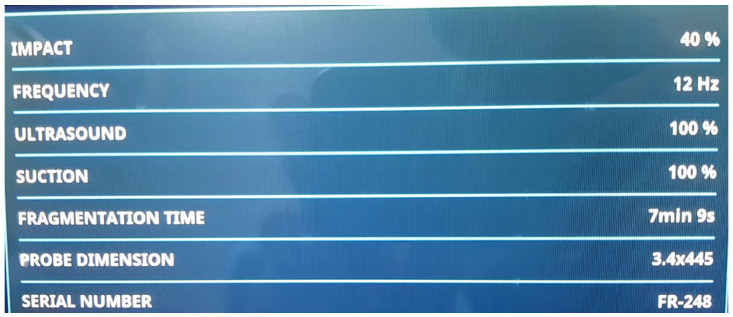
The control panel of the Trilogy device.

A total of 59 patients were included in the study, of a mean age of 54.5 (±11.8) years, a median age of 59.5 years and a range of ages between 24 to 71. In total, 38 patients (64.5%) were females and 21 (35.5%) were males. Preoperative urine cultures were positive in seven cases, and antibiotics were started before surgery, with no patient being excluded because of this reason. The mean stone diameter was 35.6 mm with a mean stone area of 419.8 ± 263 mm^2^. Mean stone density in Hounsfield units (HU) was 710.1 ± 235.5, in a range of 330.5–1299.6 mm^2^. The average number of stones was 2.08, with 12 (21.4%) complete staghorn stones and 25 (42.9%) partial staghorn stones. Guy’s stone score was 1 in 2 cases (3.6%), 2 in 19 cases (32.1%), 3 in 25 cases (42.9%) and 4 in 13 cases (21.4%). A total of 28 patients were treated with the Lithoclast Trilogy device (Figure 1) while 31 received treatment via Lithoclast Master. The Lithoclast Trilogy device parameters are listed in Figure 2. Stone features are summarized in Table 1.

A number of 13 patients presented a JJ stent (22%) inserted during previous interventions. Twelve cases (20.3%) were treated with mini PCNL with a track size of 15.9 FR while the rest of the series was treated with conventional PCNL. The dilation method utilized was Alken in 50 cases (85.7%) and Amplatz in 9 cases (14.3%). We suggest that the way we obtained access to the collecting system had no impact on any of the surgical parameters we were observing.

The Trilogy probe size was 1.5 mm for 6 patients (21.4%), 3.4 mm in 10 cases (35.7%) and 3.9 mm in 12 cases (42.9%). In three patients (10.7%), a second track was necessary. A nephrostomy tube was placed at the end of the procedure in all cases, with 24 Fr in 22 cases (78.6%) and 10 Fr in 6 cases (21.4%).

**Figure 3:**
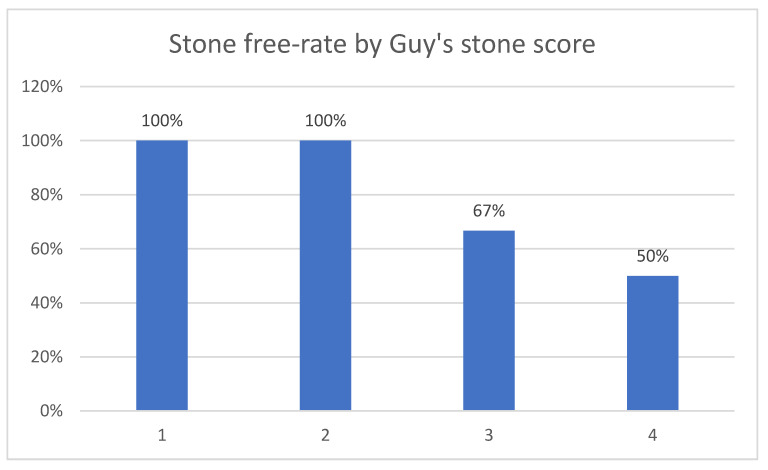
Stone free by Guy’s score in the Trilogy group.

**Figure 4:**
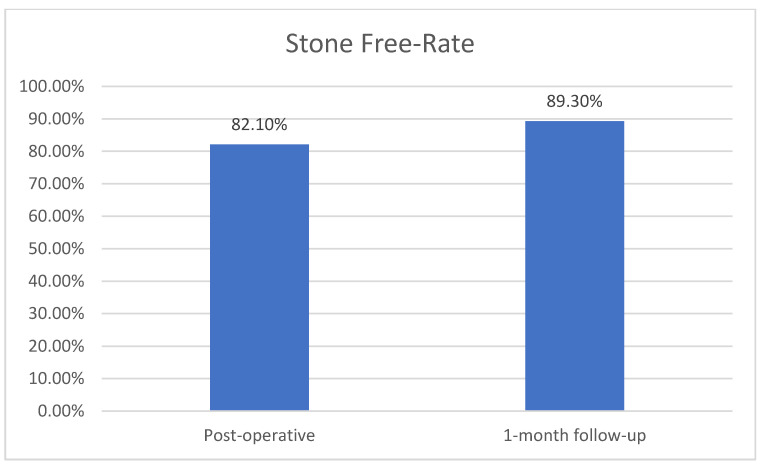
Overall stone-free rate in the Trilogy group.

**Figure 5:**
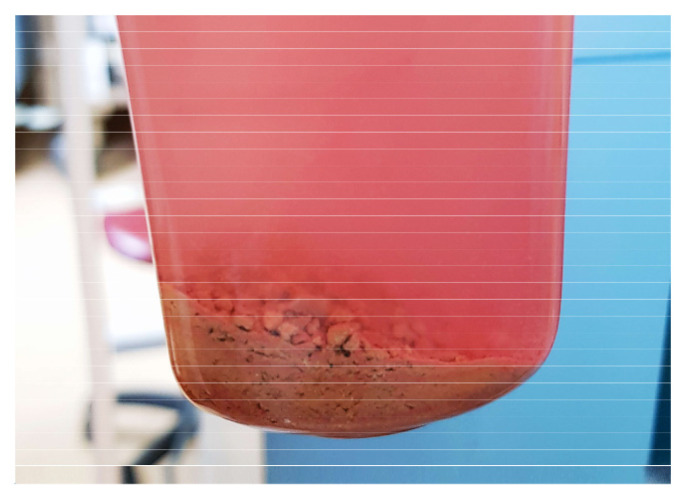
Stone fragments removed by the Trilogy device.

The mean operative time was 43.5 min, in a range from 16 to 64 min in the Trilogy group (Table 2). In the comparator group, the operative time ranged from 30 to 115 min, with a mean value of 77.13 min (Table 3). For Trilogy patients, the mean probe active time was 6.7 ± 5.3 min in a range of 0.9 to 21.3 min, and the mean stone clearance rate was 83.4 ± 62.5 mm^2^/min, with a median of 64.7 mm^2^/min. There is a significant reduction both in total operative time and probe active time, which confirms the manufacturer’s statements and our hypothesis. By not changing the probes during surgery, the total time was shorter, while combining energy modes led to faster stone fragmentation. The post-operative-stone free rates for Guy’s stone scores I, II, III and IV were 100%, 100%, 66.7% and 50%, respectively (Figure 3) while the stone free rate was 82.1% (23 of 28 patients) at the end of the procedure and 89.3% at 1-month follow-up (Figure 4). The stone aspect is illustrated in Figure 5. In the Lithoclast Master group, the stone-free rate was 71% at the end of the procedure and 78% after one month.

By looking at the comparative results, there is a very significant difference in all the parameters favoring the Trilogy device. The most important result in our opinion is the probe active time, which was almost six times shorter in the Trilogy group. We speculate that this might explain the lower complication rate in this group, by reducing the aggression imposed on the kidney in terms of time. This will probably also lead to a longer working life for the device. The stone clearance rate was about double in the Trilogy group, leading to shorter overall and intra-renal operating times.

Mean post-operative hemoglobin drop was 0.8 g/dL, with no alert values in any of the two groups. The mean creatinine rise was 0.26 mg/dL, with individual values lower after surgery, with no significant difference between the two groups. Overall complication rate in the Trilogy group was 17.9% (5 patients). Post-operative complications were classified as Clavien II—1 patient requiring blood transfusion; 1 patient with sepsis; and 1 patient with sepsis and that required blood transfusion; Clavien IIIa included 2 patients that required JJ stent after the nephrostomy tube extraction. In the comparator group, the overall complications rate, defined according to the same criteria, was 23%. Due to the very small number of cases with complications, no statistical relevance could be obtained for the comparison between the two groups in this aspect.

## 4. Discussion

The first published paper of in vitro results using begostone phantom calculi showed the superiority of LithoClast Trilogy in comparison with ShockPulse-SE and LithoClast Select [6]. A newer evaluation between different lithotripsy devices on artificial stones showed that Trilogy was more efficient per their protocol [7].

Our stone-free rates with LithoClast Trilogy for Guy’s stone scores I, II, III and IV were 100%, 100%, 66.7% and 50%, respectively. The global stone-free rate was 82.1% at the end of the procedure and 89.3% at the 1-month follow-up, which is better than the 75.7% stone-free rate obtained in the Clinical Research Office of the Endourological Society (CROES) study [8]. Sabnis et al. reported higher stone-free rates of 93% immediately post-operation and 96% after 1 month via imaging using the Trilogy device [3]. Nottingham et al. reported overall stone-free rates of 67% for LithoClast Trilogy in a similar study [4]. Our stone-free rates are higher than those of other lithotripters, per the data available in the literature, and the rates of each are as followed: ShockPulse SE—78% [9]; LithoClast Master—74% [9]; Cyberwand 56.5% [10]; LC Select—65.2% [10]; StoneBreaker—51.6% [10]. The variability of stone-free rates may be due to the different times of follow-up, imaging modality used, and stone-free rate definition, but an overview of this aspect proves the benefit of using Trilogy.

The stone clearance rate was 83.4 mm^2^/min, which is higher than that found by Nottingham et al. which was reported to be 68.9 mm^2^/min [11]. It is the only study of Trilogy which reports stone clearance efficacy by stone area. The mean stone area was higher in our study, at 419.8 mm^2^ vs. 345 mm^2^ in Nottingham et al.’s study [4]. The Trilogy device had a higher stone clearance rate compared to the lithotripters Cyberwand, LC Select or StoneBreaker (32.3 mm^2^/min, 28.9 mm^2^/min and 24.0 mm^2^/min) in all studies available to date [12]. The stone clearance rate seems to be higher for LithoClast Trilogy, but it is difficult to appreciate due to the different formulas used for the calculation of stone area. That is why a uniform system for stone burden measurement and stone clearance efficiency is necessary, as is a uniform indication of the surgical treatment of kidney calculi. Another aspect worth discussing here is the very different incidences of stone disease in different countries around the world. While there is a well-known geographical distribution difference, modern times have brought about another difference: the one between countries, based on screening programs which diagnose lithiasis proactively so that increasingly smaller size stones can be seen by urologists. Of course, if more kidney stones are diagnosed at an early stage, the utility of PCNL or miniPerc becomes less important and there are many countries reporting a significant decrease in the incidence of these surgery types [13,14]. Still, we consider that PCNL, in some form, will be around in the future so it makes a lot of sense to keep developing new technologies but also surgical skills at the same time.

Khoder et al. demonstrated Trilogy’s safety on animal tissue [5,15]. We did not have any major complications (Clavien IV–V) in our study, with only two patients requiring blood transfusion and a mean hemoglobin drop of 0.8 g/dL. Schelling analyzed a similar series, performing SWL, which led to a lower number of successful cases, while the complication rate was comparable to ours [16,17]. We did not have any device-related problems, malfunctions, or failures. Compared to the literature, this data seems a bit too optimistic, but similar results have been reported by other scholars [18,19,20].

A recent meta-analysis looks at the combined approach to the kidney, using PCNL and retrograde ureteroscopy for complex renal stones. The authors conclude that this novel technique brings better stone free rates and fewer complications and requires less blood transfusions compared to PCNL alone. The devices used for lithotripsy are not compared and no comment is made in this direction [21].

The main limitation of this study is the relatively low number of patients, and the high gender imbalance leading to the number of females being almost double that of males. The comparator device uses only one type of energy, so our study analyzes slightly different devices, due to Trilogy not having a competitor with similar features from a different producer. In general, prospective, randomized surgical studies are difficult to conduct, mainly because patients need to be informed about the surgical procedure they are about to undergo; our study did not compare different surgical techniques but only two different devices used for the same purpose, so this was more easily accepted by the ethics committee and the patient. Future studies will become available and contribute to the validation of this device through systematic reviews and meta-analyses [22].

## 5. Conclusions

In our experience, Swiss LithoClast^®^ Trilogy, a device that combines ultrasonic and ballistic energy, is a safe and effective method of lithotripsy for PCNL and miniPerc. Our stone-free rate and stone clearance rate were significantly higher when using this technology compared to those found using LithoClast Master, while the mean operative time decreased, and the complication rate remained low. We conclude that this novel device can achieve the goal of reducing complication rates and operative times for treating kidney stones, marking a much-needed step forward in improving minimally invasive kidney surgery.

More studies and comparisons are needed in order to gain enough clinical evidence to support the superiority of Trilogy, and we plan to keep acquiring data in our department and present it in another future paper.

## Figures and Tables

**Table 1:** Patient and stone characteristics.

Characteristics		Range
Mean age (years)	54.5 ± 11.8	24–71
Gender		
Male	21 (35.5%)	
Female	38 (64.5%)	
Mean stone area(mm^2^)	419.8 ± 263	93.6–1142.5
Mean stone density (HU)	710.1 ± 235.5	330.5–1299.6
Guy’s stone score		
1	2 (3.6%)	
2	19 (32.1%)	
3	25 (42.9%)	
4	13 (21.4%)	
No. of stones		
1	26 (42.9%)	
2	17 (28.6%)	
>2	16 (28.5%)	
Main stone location		
Renal pelvis	17 (28.6%)	
LP	4 (7.1%)	
Partial staghorn	25 (42.9%)	
Staghorn	13 (21.4%)	

**Table 2:** Intervention times and clearance rates in the Trilogy group.

Characteristics	Mean	Median	Range
Total intervention time (min)	43.5 ± 11.24	44	16–64
Nephroscopy time (min)	35 ± 11.8	33	10–58
Probe active time (min)	6.7 ± 5.3	6	0.9–21.3
Stone clearance rate (mm^2^/min)	83.4 ± 62.5	64.7	14.6–298.7

**Table 3:** Comparison of main surgical parameters between the two groups.

	Trilogy	Lithoclast Master	*p*-Value
Total intervention time (min)	43.5 ± 11.24	77.13 ± 19.14	*p* < 0.005
Nephroscopy time (min)	35 ± 11.8	68.6 ± 16.8	*P* < 0.005
Probe active time (min)	6.7 ± 5.3	39 ± 11.4	*p* < 0.005
Stone clearance rate (mm^2^/min)	83.4 ± 62.5	35.7 ± 15.8	*p* < 0.005

## Data Availability

Not applicable.
